# Knee Osteoarthritis Treatment with the KineSpring Knee Implant System: A Report of Two Cases

**DOI:** 10.1155/2012/297326

**Published:** 2012-12-06

**Authors:** David A. Hayes, Larry E. Miller, Jon E. Block

**Affiliations:** ^1^Brisbane Orthopedic and Sports Medicine Center, Level 5, 259 Wickham Terrace, Brisbane, QLD 4000, Australia; ^2^Miller Scientific Consulting, 26 Portobello Road, Arden, NC 28704, USA; ^3^The Jon Block Group, 2210 Jackson Street, Suite 401, San Francisco, CA 94115, USA

## Abstract

Osteoarthritis (OA) is a leading cause of disability in middle-aged and older adults with the prevalence expected to increase by 40% by 2025. This dramatic projected increase in OA reflects, in large part, the alarming obesity epidemic. Indeed, it is now well understood that abnormal loading across the knee joint due to malalignment and/or excessive weight gain is responsible for accelerating OA progression. Consequently, there is a therapeutic need for alternative knee OA treatments that directly address joint overload to fill the gap between ineffective conservative care and invasive joint-modifying surgical procedures. We describe two cases that presented with bilateral knee OA resistant to conservative treatments, each with one knee previously and unsuccessfully treated with high tibial osteotomy to improve alignment and the contralateral knee successfully treated with a joint-preserving, load-absorbing implant (KineSpring Knee Implant System).

## 1. Introduction

Osteoarthritis (OA) is a progressive disease that is characterized by joint pain and dysfunction secondary to articular cartilage loss [1]. Osteoarthritis is a leading cause of disability in middle-aged and older adults [2], and the prevalence is expected to increase by 40% by 2025 [3, 4]. Much of this projected increase is due to the soaring rates of obesity as excessive weight gain has been shown to accelerate the progression of OA by abnormally loading the knee joint [5]. Despite the wide range of treatment options available to the patient with knee OA, each suffers from distinct limitations. Conservative treatments such as activity modification, weight loss, physical therapy, orthotics, and/or bracing are initially recommended for mild knee OA. If symptoms worsen or become chronic despite conservative care, antiinflammatory and/or analgesic medications, intra-articular hyaluronic acid and/or steroid injections, and arthroscopic lavage and debridement may be attempted. While these treatment options may provide short-term pain palliation in some patients, they do not address abnormal joint loading, a primary cause of disease progression [5–8]. Total knee arthroplasty (TKA) represents the gold standard treatment for end-stage knee OA, although unicompartmental knee arthroplasty (UKA) or high tibial osteotomy (HTO) may be considered in selected patients with single-compartment disease. The success of these surgical modalities, however, requires careful consideration of the risk of serious complications and significant recovery periods for patients [9]. There is a distinct therapeutic need for alternative knee OA treatments that directly address joint overload to fill the gap between ineffective conservative care and joint-modifying surgical procedures [10]. Two patients presented with bilateral knee OA resistant to conservative treatments, each with one knee previously and unsuccessfully treated with HTO and the contralateral knee successfully treated with a joint-preserving, load-absorbing implant.

## 2. Case Report

### 2.1. Device

**Figure 1:**
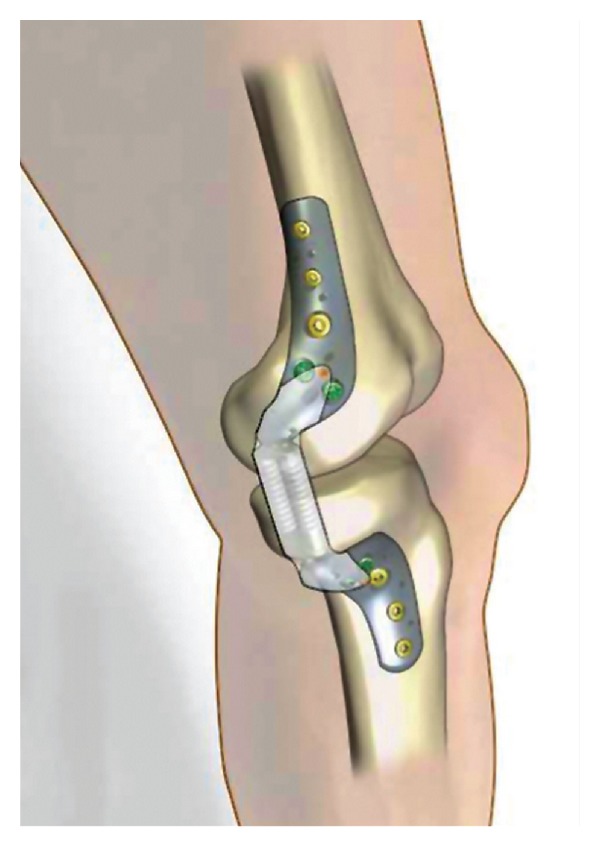
Medial view of the KineSpring Knee Implant System.

**Figure 2:**
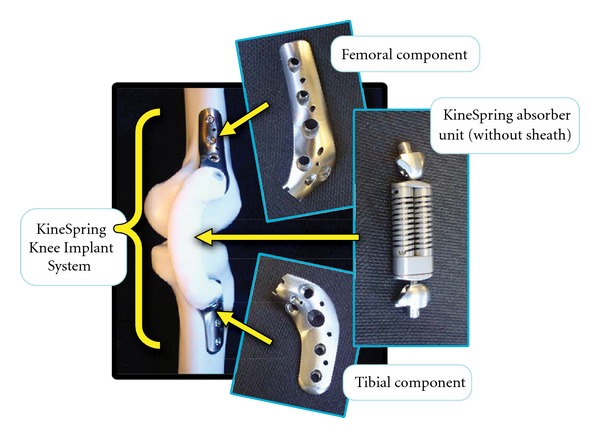
Components of the KineSpring Knee Implant System.

The KineSpring Knee Implant System (Moximed, Inc., Hayward, CA, USA) (Figure 1) is a knee prosthesis consisting of titanium alloy femoral and tibial bases and a covered cobalt/cobalt chrome alloy absorber specifically designed to reduce load at the medial compartment of the knee joint during the stance phase of gait (Figure 2). The KineSpring System absorbs a maximum load of 40 pounds during full knee extension, which is comparable to typical knee adduction moments. Consequently, chronic medial compartment loading is reduced without significant increases in lateral compartment loading [11]. This magnitude of unloading has been shown to yield clinically meaningful improvements in joint pain and function in OA patients [12]. The device incorporates two highly polished metal ball-and-socket joints that allow natural knee range of motion, including unlimited internal-external rotation, 50 degrees of varus-valgus angulation, and 155 degrees of flexion-extension movement.

### 2.2. Procedure

The procedure is performed under general anesthesia with the patient in the supine position. Initial access is achieved via a 3-4 inch incision proximal to the knee. The femoral base is then attached to the medial distal femoral cortex. Next, a second incision is made just distal to the knee where the absorber and tibial base assembly are positioned subcutaneously and attached to the prepositioned femoral base proximally and the medial proximal tibial cortex distally. The compressible absorber resides on the medial side of the knee within the adjacent subcutaneous tissue. Activation of the absorber is completed before wound closure. Importantly, the procedure is performed entirely outside of the joint capsule with minimal disruption of the knee anatomy, requiring no resection of bone, cartilage, or ligament. Patients are encouraged to ambulate immediately following recovery from anesthesia.

### 2.3. Case 1

**Figure 3:**
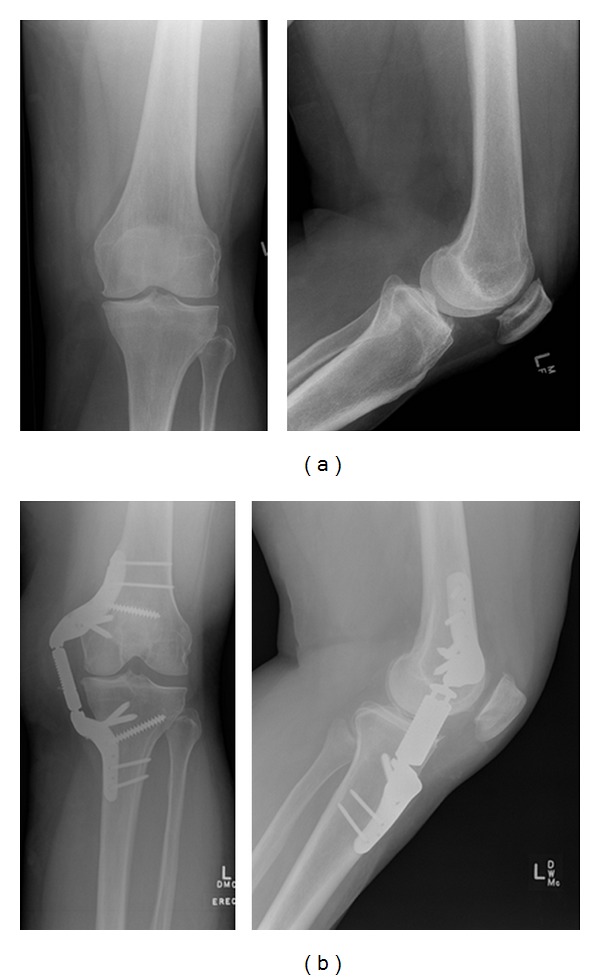
Anterior (left) and medial (right) radiographic view of the left knee at pretreatment (a) and at 3 years demonstrating the KineSpring System *in situ* (b).

A 51-year-old female presented to the clinic s/p HTO on the right knee and with severe left-knee OA-related pain and dysfunction (K-L grade 2) of 2-year duration that was unresponsive to maximal conservative treatment. The patient was dissatisfied with the invasiveness of the procedure, prolonged recovery, and potential for compromised TKA outcomes provided by the initial HTO and elected not to repeat the procedure on the contralateral knee. She was treated with the KineSpring System and returned for regular followup visits including imaging through 3 years (Figure 3).

### 2.4. Case 2

A 53-year-old obese (BMI: 39 kg/m^2^) male presented s/p HTO on the left knee and with severe right knee OA-related pain and dysfunction (K-L grade 1) of 1-year duration despite activity modification, physical therapy, and maximum pharmacological management. The patient was dissatisfied with the invasiveness of the procedure, prolonged recovery, and potential for compromised TKA outcomes provided by the initial HTO and elected not to repeat the procedure on the contralateral knee. The patient was treated with the KineSpring System at our clinic and was followed for 1 year.

## 3. Results

### 3.1. Case Number 1

No device-related complications were reported during the procedure or in followup. At the 3-year visit, all patient-reported outcomes were significantly improved compared to baseline with WOMAC pain score improving by 90% (50 to 5), WOMAC Function score improving by 76% (41 to 10), WOMAC Stiffness score improving by 100% (50 to 0), KSS Knee score improving by 57% (60 to 94), and KSS Function improving by 27% (79 to 100).

### 3.2. Case Number 2

**Figure 4:**
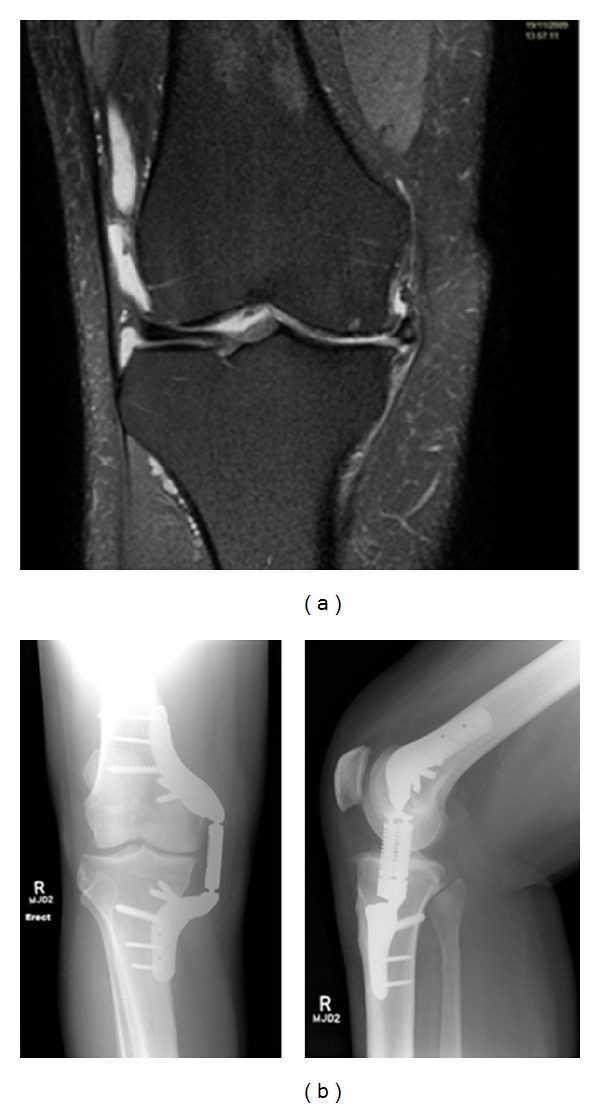
(a) Anterior view of the right knee with pre-treatment magnetic resonance imaging (a) and anterior (left) and medial (right) 1-year radiographs demonstrating the KineSpring System *in situ* (b).

The patient experienced no procedural complications, and no device-related complications or adverse radiographic findings have been reported through 1 year (Figure 4). At the 1-year followup visit, WOMAC pain score improved by 38% (40 to 25), WOMAC Function score improved by 43% (44 to 25), WOMAC Stiffness score improved by 50% (50 to 25), KSS Knee score improved by 42% (62 to 88), and KSS Function score improved by 138% (40 to 95).

## 4. Discussion

Joint preserving implants are used in orthopedics with increasing frequency in order to offer patients safer treatment alternatives to surgery without sacrificing clinical effectiveness. Currently, treatment options for the patient with knee OA include conservative care, HTO, or UKA/TKA. Unfortunately, no viable joint preserving device is available for knee OA with an acceptable safety and effectiveness profile. The ideal characteristics of such a treatment would include excellent patient acceptance, effectiveness in reducing pain and improving function without compromising the integrity of the lateral or patellofemoral compartments, and an easily reversible procedure that maintains the anatomical integrity of the knee joint.

Our initial experience with the KineSpring System fulfilled all of these criteria. Patient acceptance of the device was excellent, which can be attributed to the fact that it resides in subcutaneous tissue, preserves the normal knee joint range of motion, and offers the potential for a short recovery time. Knee joint pain and function were improved by a clinically meaningful margin in both patients based on validated, knee-specific questionnaires. Finally, since all surrounding anatomical structures (e.g., bone, muscles, and ligaments) remain intact with the procedure, the KineSpring System may be explanted, if needed, via the same access route without compromising future surgical options.

The concept behind the KineSpring System mechanism of action is sound since excessive and/or chronic joint loading is believed to be the primary risk factor in knee OA development and progression [5]. Based on the prevailing premise that cumulative adverse loading at the knee joint is a strong predictor of OA [13–15], therapies designed to reduce chronic knee loading may halt the progression of OA [16, 17] and may even allow healing [18].

The novelty of the cases presented is that each patient suffered from bilateral knee OA, with one knee previously treated with HTO and the contralateral knee subsequently treated with the KineSpring System. Each patient opted for KineSpring implant based on dissatisfaction with previous contralateral HTO including lingering mild to moderate pain as well as strong reluctance to undergo TKA. Unlike the KineSpring procedure, HTO involves significant bone removal and reshaping in an effort to shift loading from the diseased compartment to the unaffected compartment. The surgical invasiveness of HTO has been shown to require approximately 9 days of postoperative hospitalization, on average [19] as well as a substantial delay in return to work typically lasting 3 months with some patients incapacitated far longer [20]. Additionally, despite satisfactory early outcomes with HTO, patient outcomes reliably worsen over time [21]. Significant procedural risks also limit the utility of HTO, which include infection (2–55%), deep vein thrombosis (1–10%), delayed or nonunion (0–14%), and peroneal nerve injury (0–20%) [21].

Recent estimates suggest that over 4 million Americans suffer from knee OA such that the ability of ambulate is compromised [10, 22]. However, only 500,000 knee arthroplasties and HTOs are performed annually in the United States, representing only 13% of all patients with debilitating symptoms [23]. These data highlight the distinct therapeutic void for knee OA treatments with an appreciable percentage of patients lingering in the treatment gap, often for many years [10]. Implants such as the KineSpring System have great potential to fill this void by offering the possibility of a safe and effective joint-sparing treatment that can be revised subsequently if necessary.

The KineSpring System is indicated for patients with symptomatic medial knee OA. The KineSpring System is not appropriate in patients with moderate to severe osteoporosis, symptomatic lateral OA in the affected knee, or varus alignment >10 degrees in the affected knee. Despite the excellent patient outcomes demonstrated in this case series, additional clinical trials with the KineSpring System are underway that will further the evidence for the role of this device in treating medial compartment knee OA.

In conclusion, the KineSpring System is a promising joint unloading therapy for patients with symptomatic medial compartment knee OA that offers advantages of early ambulation, maintenance of knee joint anatomic integrity, and medial compartment unloading resulting in symptom relief and improved knee function.
